# The Potential of Vouacapanes from *Pterodon emarginatus* Vogel Against COVID-19 Cytokine Storm

**DOI:** 10.34172/apb.2023.016

**Published:** 2021-10-10

**Authors:** Leandra de Almeida Ribeiro Oliveira, Arthur Christian Garcia da Silva, Douglas Vieira Thomaz, Fabiana Brandão, Edemilson Cardoso da Conceição, Marize Campos Valadares, Maria Tereza Freitas Bara, Dâmaris Silveira

**Affiliations:** ^1^Faculty of Pharmacy, Federal University of Goiás, P.O. Box 131, Goiânia, GO, Brazil.; ^2^Faculty of Health Sciences, University of Brasilia, Campus Darcy Ribeiro, Asa Norte, 70910-000, DF, Brazil.

**Keywords:** Coronavirus, Furanoditerpene, Inflammatory cytokines, COVID-19, Sucupira

## Abstract

***Purpose:*** The emergence of the COVID-19 pandemic has led to the search for potential therapeutic responses for various aspects of this disease. Fruits of *Pterodon emarginatus* Vogel (Fabaceae), sucupira, have been used in Brazilian traditional medicine because of their anti-inflammatory properties, which have been proven *in vivo, in vitro*, and *in silico*. Therefore, the aim of this work is to evaluate *P. emarginatus* oleoresin and isolated diterpenes by *in vitro* anti-inflammatory models.

***Methods:*** In this study, the mechanisms underlying the anti-inflammatory activity of *P. emarginatus* oleoresin and vouacapanes 6α,19β-diacetoxy-7β,14β-dihydroxyvouacapan (V1), 6α-acetoxy-7β,14β-dihydroxyvouacapan (V2), and methyl 6α-acetoxy-7β-hydroxyvouacapan-17β-oate (V3) were investigated in HaCaT cells.

***Results:*** Oleoresin, V2, and V3 inhibited phospholipase A2 (30.78%, 24.96%, and 77.64%, respectively). Both vouacapanes also inhibited the expression of COX-2 (28.3% and 33.17%, respectively). The production of interleukin 6 (IL-6) was inhibited by oleoresin by 35.47%. However, oleoresin did not interfere with Nrf-2 expression or IL-8 production.

***Conclusion:*** The results support the ethnomedicinal use of *P. emarginatus* oleoresin as an anti-inflammatory herbal medicine, and also highlight *P. emarginatus* oleoresin and isolated vouacapanes as an attractive therapeutic approach for COVID-19 through the reduction or chronological control of the inflammatory mediators IL-6, cyclooxygenase-2 (COX-2), phospholipase A2, and INF-y (indirectly) during the SARS-CoV-2 infection process.

## Introduction

 Since the first reports of unexplained respiratory infections in Wuhan, China, at the end of December 2019, the world has faced one of the worst pandemic crises. From an initial 4000 reported deaths in China, the fatal cases of SAR-CoV-2 infection jumped to more than 4.28 million worldwide by mid-August 2021.^1^ In Brazil, the situation is deeply worrying, with 20 416 183 confirmed infections and more than 570 000 fatal cases, in mid-August 2021.^2,3^

 COVID-19, caused by SAR-CoV-2, proved to be more than a severe respiratory infection; it involves a number of symptoms, from cough to cardiac and central nervous system (CNS) manifestations.^4,5^

 SARS-CoV-2 infection develops a physiopathology comparable to that of SARS-CoV. This infection results in an aggressive inflammatory response provoking damage to the airways,^6^ and may lead to an exacerbated signaling mediated by cytokines from the immune system, ending in a phenotype named “cytokine storm”.^5^ This can result in death in about 28% of COVID-19 cases,^5^ since the “cytokine storm syndromes” correspond to hypercytokinemia and a hyperinflammatory process. An increase in interleukins such as monocyte chemoattractant protein 1, macrophage inflammatory protein 1-α, granulocyte-colony stimulating factor, interferon-γ (INF-γ) inducible protein 10, and tumor necrosis factor,^7,8^ are observed, leading to a lethal and fulminating outcome.^9^

 However, the assumption that COVID-19 ends with the symptoms, and the avoidance of mortality, have subsided with increasing reports of persistent and prolonged effects, recognized as post-COVID-19 (or post-acute-COVID-19) syndrome.^10,11^

 Upon entering the host cells, the virus needs the surface receptors angiotensin-converting enzyme 2 (ACE2),^12^ and TMPRSS2.^13^ Even though ACE2 is significantly expressed in the type II alveolar cells of the lungs, studies have demonstrated levels of SARS-CoV-2 receptor expression in different tissues and cells, such as the upper esophagus, stratified epithelial cells, kidney cells, urothelial cells, enterocytes, and cardiomyocytes, which supports cellular outspreading of SARS-CoV-2 infection.^14^ Consequently, COVID-19 is not only limited to respiratory disorders but also to kidney, liver, heart, and gastrointestinal tract illnesses.^15^ Interestingly, ACE2 expression is significantly higher in keratinocytes,^16^ suggesting that this kind of cell can be infected.

 Once inside the cells, the productive replication and discharge of the new virus undergo an inflammatory process due to the release and recognition of damage-associated molecular patterns. The details of the infection and hyper-inflammation processes have recently been elucidated. In the lung, alveolar macrophages signal pro-inflammatory cytokines and chemokines, including interleukin 6 (IL-6), phospholipases A2, cyclooxygenase-2 (COX-2), and IFN-γ. These messengers attract immune system cells to the site of infection, promoting inflammation and a pro-inflammatory feedback loop. The accumulation of immune cells in the lungs causes the overproduction of pro-inflammatory cytokines, which eventually damages the lung infrastructure. The resulting cytokine storm may spread to other organs, causing multi-organ damage.^8^

 Research groups are endeavoring to find drugs to treat COVID-19, and in addition to vaccines, already used drugs, natural products, and traditional medicines (mainly Chinese) have been trialed.^17-20^ However, only a few promising agents have been found to treat infections caused by SARS-CoV-2.^21^

 Brazil’s biodiversity has been claimed to be a potential source of new drugs. With more than 40 000 native plant species distributed in several biomes,^22^ the traditional use of herbal medicines is prevalent throughout the country. *Pterodon emarginatus* Vogel (Fabaceae) is a native species largely used in traditional medicine, and “sucupira” fruits are available at the Brazilian medicinal flora market.^23^ This species was included in the manuscripts and publications of George Gardner (1812–1849), and Saint-Hillaire (1779-1853),^24,25^ which described the use of the essential oil of *Pterodon* fruits for toothaches. Several ethnomedicinal surveys around Brazil have highlighted the use of sucupira as an anti-inflammatory remedy. In the Southeast region, hydroalcoholic “garrafada” (a traditional maceration, usually with cachaça as solvent) of *Pterodon* fruits has been used in popular medicine for inflammation, mainly in cases of rheumatism, sore throat, bronchitis, and asthma^26^; in the Northeast region, a decoction of *Pterodon* fruits has been used for its anti-inflammatory and depurative properties.^27^

 The chemistry of* P. emarginatus* has been well established. Isoflavones and diterpenes (vouacapane-type) are the main components of oleoresin.^28-34^ Vouacapane diterpenoids are low-to-medium polarity secondary metabolites. Studies suggested the vouacapane skeleton as the main pharmacophore, supported by pharmacological investigations of isolated compounds, as well as *P*.*emarginatus* oleoresin-derived vouacapane diterpenoids.^32^ Chemoinformatic and *in vivo* investigations suggested the thermodynamic feasibility of *P*. *emarginatus*-derived vouacapane diterpenoid docking in both human and murine COX-2,^28^ as well as a possible impairment of pro-inflammatory mediators from the prostaglandin E2 (PGE2) pathway,^35^ which might indicate the involvement of diterpenoid derivatives in the reported *P*.*emarginatus* oleoresin antinociceptive and anti-inflammatory action.^36^

**Figure 1:**
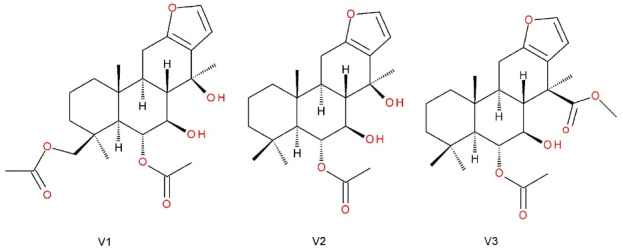
Vouacapanes isolated from *Pterodon emarginatus* Vogel (Fabaceae).6α,19β-diacetoxy-7β,14β-dihydroxyvouacapan (V1), 6α-acetoxy-7β,14β-dihydroxyvouacapan (V2), and methyl 6α-acetoxy-7β-hydroxyvouacapan-17β-oate (V3)

 Given the importance of elucidating the pharmacodynamics of *P*.*emarginatus* products, the anti-inflammatory actions of oleoresin and vouacapanes 6α,19β-diacetoxy-7β,14β-dihydroxyvouacapan (V1), 6α-acetoxy-7β,14β-dihydroxyvouacapan (V2), and methyl 6α-acetoxy-7β-hydroxyvouacapan-17β-oate (V3) (Figure 1) were evaluated in HaCaT human keratinocytes exposed to UVA radiation or 5-fluorouracil (5-FU). Therefore, different targets were evaluated, including modulation of COX-2, NRF2, and IL-6, as well as the production of IL-8, IL-1β, IL-10, tumor necrosis factor (TNF), and IL-12p70. The inhibition of phospholipase A2 by oleoresin and the three isolated vouacapanes were also evaluated.

## Materials and Methods

###  Reagents and solutions

 Dulbecco’s modified Eagle’s medium (DMEM), nutrient mixture F-12 (Ham’s F-12), 5-fluorouracil (5-FU), 3-[4,5-dimethylthiazol-2-yl]-2,5-diphenyl tetrazolium bromide (MTT), 2,7-dichlorofluorescein diacetate (DCFH-DA), Triton X-100, protease inhibitor cocktail, bicinchoninic acid protein assay kit, bovine serum albumin (BSA), and Hanks’ balanced salt solution (HBSS) were purchased from Sigma-Aldrich (St. Louis, USA). BD Cytofix/Cytoperm^TM^ solution and BD cytometric bead array (CBA) human inflammatory cytokine kit (catalog no. 551811) were obtained from BD Biosciences (San Jose, USA). Fetal bovine serum (FBS), penicillin/streptomycin solution, Hoechst 33 342, and TrypLE^TM^ expression were acquired from Invitrogen/Life Technologies (Carlsbad, USA). Dimethyl sulfoxide (DMSO) and Tween-20 were obtained from Vetec (Rio de Janeiro, Brazil). Alexa Fluor 488-conjugated anti-human nuclear factor erythroid 2–related factor 2 (NRF2) (EP1808Y) antibody was acquired from Abcam Plc (Cambridge, United Kingdom). Phycoerythrin (PE)-conjugated anti-COX-2 (sc-7951) was acquired from Santa Cruz Biotechnology (Santa Cruz, CA, USA).

###  Plant material

 Fruits of *P*.*emarginatus* Vogel (Fabaceae) were collected in Bela Vista de Goiás, Brazil in September 2007. A voucher was deposited at the Federal University of Goias (UFG) herbarium (number 27 155).

 The oleoresin was extracted by cold pressing in a continuous mini-press (MPE-40 ECIRTEC, Bauru, São Paulo, Brazil), as previously described,^30^ with a yield of 30% weight. The oleoresin (OR) was stored at -20°C until analysis. The isolation of vouacapanes 6α,19β-diacetoxy-7β,14β-dihydroxyvouacapan (V1), 6α-acetoxy-7β,14β-dihydroxyvouacapan (V2) and 6α-acetoxy-7β-hydroxyvouacapan-17β-oate (V3), as well as the structural elucidation, were also described in previous work.^30,32^ For all *in vitro* evaluations, the concentration used was 7.5 µg/mL (oleoresin), 19 µg/mL (V1), 13.8 µg/mL (V2) and 4 µg/mL (V3), defined according to the Cell Viability 90% concentration (CV_90_) of each evaluated substance, determined by MTT cytotoxicity assay.^37^

###  Cell culture

 HaCaT human immortalized keratinocytes were acquired from the Rio de Janeiro Cell Bank (Rio de Janeiro, RJ, Brazil). HaCaT cells were cultured in DMEM supplemented with 10% heat-inactivated FBS, penicillin (100 IU/mL), and streptomycin (100 μg/mL). Cells were cultivated in an incubator (Thermo Scientific Revco CO_2_ incubator, Waltham, USA) at 37°C in a humidified atmosphere of 5% CO_2_. The cells were harvested using TrypLE^TM^ Express solution when they reached approximately 70% confluence. Cell number and viability were determined using the Trypan Blue exclusion method, employing the TC20^TM^ automated cell counter (Hercules, CA, USA), according to the manufacturer’s instructions. Experiments were conducted when cell viability values were higher than 90%.^37^

###  5-FU preparation 

 A stock solution of 5-FU (10 mg/mL) was prepared in DMSO according to established protocols,^36^ and kept in an ultrasonic bath for 10 minutes at room temperature. The stock solution was stored at -20°C and thawed immediately before use. For the cell-based assays, the 5-FU solution was diluted in complete medium so that the final DMSO concentration did not exceed 0.4% (v/v).

###  Cytotoxicity assessment

 The cytotoxicity of oleoresin and vouacapanes on HaCaT cells was evaluated using an MTT reduction assay. Briefly, HaCaT cells were seeded in 96-well plates (1.5 × 10^4^ cells/well) and cultivated overnight for adhesion. After that, cells were exposed to decreasing concentrations of oleoresin and V1, V2, and V3 compounds (125–0.98 µg/mL) for 24 hours. Then, the supernatant was discarded, and the cells were washed with 150 µL/well of phosphate buffered saline (PBS). Afterward, 100 µL of MTT solution prepared in DMEM (0.5 mg/mL) were added per well. The cells were then incubated for 3 h. Finally, the supernatant was discarded, and the formazan crystals formed were solubilized in 100 µL/well of DMSO under agitation, and the absorbance of the wells was determined using a spectrophotometer plate reader (Multiskan Spectrum, Thermo Scientific, MA, USA) at 560 nm. The experiments were conducted in triplicate, and cell viability was determined in comparison to the absorbance of the negative control (untreated cells).

###  In vitro expression of COX-2 and NRF2

 COX-2 and NRF2 activity assays were conducted according to standard protocol.^28^ Briefly, the test consisted of seeding HaCaT cells into 6-well plates (2.0 × 10^5^ cells/well) and incubated overnight. Cells were then pre-treated with 1 mL/well of either oleoresin, V1, V2, and V3 for 24 hours, using the mentioned concentrations. 5-FU (10 μg/mL) was added to each well, and the culture was incubated for an additional 24 hours. Cells were then collected using TrypLE^TM^ Express solution, washed twice with PBS-BSA (0.1%, w/v), and centrifuged at 1500 rpm at 25°C for 5 minutes. Cells were then incubated with BD Cytofix/Cytoperm^TM^ solution at 4°C for 20 minutes and then washed twice with PBS-Tween 20 (0.05, v/v). Cells were incubated with specific monoclonal antibodies (anti-NRF2 or anti-COX-2) and protected from light for 30 minutes at room temperature. The cells were again washed twice with PBS-T20, centrifuged at 1500 rpm, 25°C for 5 minutes, and suspended in 200 μL PBS for flow cytometry analysis.^37^

###  Cytokine measurement in HaCaT cells exposed to UVA radiation

 HaCaT cells (7.5 × 10^4^ cells/well) were seeded into 24-well culture plates and incubated overnight for adhesion. The cells were then pre-treated with oleoresin, V1, V2, or V3 (4 µg/mL) for 24 hours. Later, the supernatant was discarded, and the wells were rinsed with 1 mL of PBS solution, and 500 µL of Hanks’ Balanced Salts Solution was added to each well. The assays were conducted in two plates, one protected from UVA radiation, and the other was subjected to 20 J/cm^2^ at Caron’s Photo-Stability Chamber. The cells were then treated with either oleoresin, V1, V2, or V3, and incubated for 12 h. After incubation, cells were washed twice with PBS (1 mL/well), and cell lysates were obtained using 250 μL/well of PBS solution containing 0.5% (v/v) Triton X-100 and protease inhibitor cocktail. Cell lysates were stored at −80°C until analysis. Cytokine (TNF, IL-1β, IL- 6, IL-8, IL-10, and IL-12p70) levels were measured using the CBA method, using the BD CBA human inflammatory cytokine kit (BD Biosciences), according to the manufacturer’s instructions, using a flow cytometer (BD FACSCanto II, BD Biosciences, San Jose, CA, USA). The level of cytokine (pg) was expressed as a ratio of total protein content (mg), determined with a bicinchoninic acid protein assay kit (Sigma-Aldrich, St. Louis, USA) using BSA as a standard, in accordance with the manufacturer’s instructions.^37^

###  In vitro inhibition of phospholipase A2

 The inhibitory activity towards phospholipase A2 was determined using the EnzChek Phospholipase A2 Assay kit (Invitrogen). The manufacturer’s protocol was followed. Samples (oleoresin, V1, V2, and V3) were in the same concentrations evaluated in the cell-based experiments and were incubated for 10 min at 25°C. Phospholipase A2 was used as the positive control, while DMSO 0.15% was used as the negative control. All assays were conducted in triplicates.

###  Statistics analysis

 The data are expressed as the mean ± standard error. Group comparisons were conducted using the Student’s *t* test. Statistical significance was set at *P* < 0.05. For the cytotoxicity tests, the IC_50_ and CV_90 _values were obtained using non-linear regression.

## Results and Discussion

###  Cell viability towards P. emarginatus oleoresin and vouacapane diterpenoids

**Figure 2:**
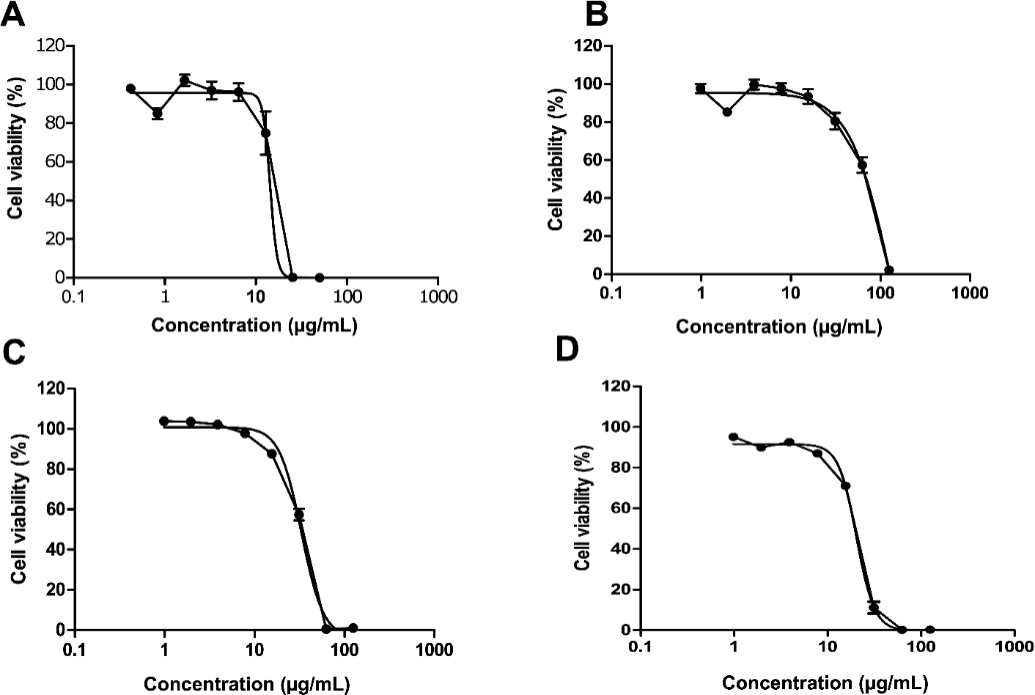
HaCaT cell viability after exposure to *P*. *emarginatus* oleoresin (A), V1 (B), V2 (C), V3 (D).Cells were exposed to decreasing concentrations of oleoresin and V1, V2, and V3 compounds (125 – 0.98 µg/mL) for 24 hours, and cell viability was determined using the MTT reduction assay

 To preliminarily evaluate the cytotoxicity of oleoresin and vouacapane diterpenoids, an MTT reduction assay was performed (Figure 2).

 Results showed that oleoresin, V1, V2 and V3 were cytotoxic in a concentration-dependent manner, and the half-maximal inhibitory concentration (IC_50_) values obtained were 28.1 µg/mL, 70.0 µg/mL, 33.2 µg/mL and 20.4 µg/mL, respectively. In parallel, the cell viability 90% (CV_90_) was determined for each tested sample (oleoresin, V1, V2 or V3) as 7.5 μg/mL, 19.0 μg/mL, 13.8 μg/mL and 4.0 μg/mL, respectively (Figure 2A, 2B, 2C, and 2D).

 Regarding the toxicity of vouacapane diterpenoids, the literature reports that some cell lineages, such as murine cells (3T3), are susceptible to damage upon their administration at IC_50_ ranging from 63.0 to 95.2 nmol/mL (*i.e.,* 22.83 µg/mL to 34.33 µg/mL),^38^ which nonetheless implies the importance of a preliminary cytotoxicity investigation in order to proceed with pharmacological and immunologic studies.^32,39,40^

###  Anti-inflammatory investigation of P. emarginatus oleoresin and vouacapane diterpenoids

####  In vitro expression of COX-2 and NRF2

**Figure 3:**
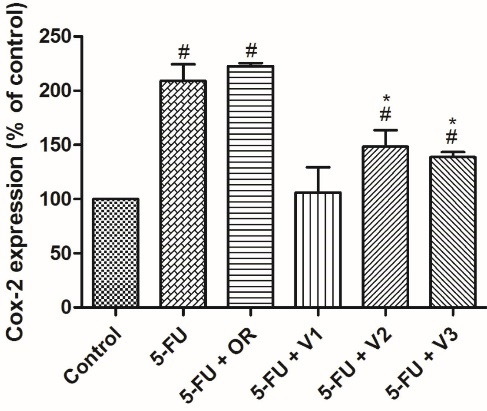
Effects of *Pterodon emarginatus* oleoresin (OR) or isolated vouacapanes on the expression of COX-2. HaCaT cells were pretreated with OR (7.5 µg/mL), V1 (19 µg/mL), V2 (13.8 µg/mL) or V3 (4 µg/mL) for 24 h. After that, cells were treated with the same samples cited above and concomitantly exposed to 5-FU (10 μg/mL) for an additional 24 h. Cells were then analyzed by flow cytometry. Each bar represents the mean ± SD of three independent experiments. (^#^*P* < 0.05 vs control, * P < 0.05 vs. 5-FU)

**Figure 4:**
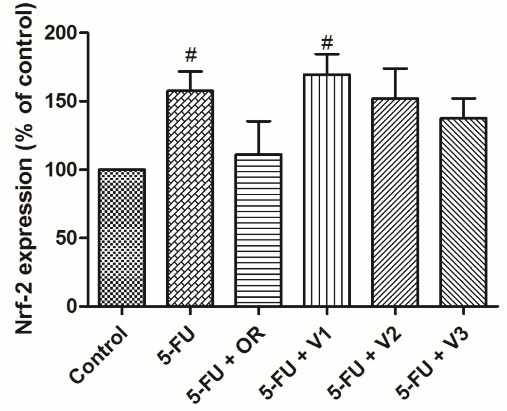
Effects of *Pterodon emarginatus* oleoresin (OR) or isolated vouacapanes on the expression of Nrf-2. HaCaT cells were pretreated with OR (7.5 µg/mL), V1 (19 µg/mL), V2 (13.8 µg/mL) or V3 (4 µg/mL) for 24 h. After that, cells were treated with the same samples cited above and concomitantly exposed to 5-FU (10 μg/mL) for an additional 24 h. Cells were then analyzed by flow cytometry. Each bar represents the mean ± SD of three independent experiments. (^#^*P* < 0.05 vs control)

 COX-2 is an important enzyme involved in prostanoid biosynthesis. Its expression is intimately related to the onset as well as the inflammatory response development.^41,42^ On the other hand, NRF2 is a transcription factor activated in response to oxidative stress conditions, which can trigger inflammatory alterations, and it is an important contributor to this process since it promotes the recruitment of inflammatory cells and regulates the NLRP3 inflammasome.^43^ Therefore, *P*.*emarginatus* oleoresin and its main vouacapane diterpenoids were investigated for their influence on the cellular expression of COX-2 and NRF2 (Figures 3 and 4).

 As can be seen in Figure 3, vouacapanes V2 and V3 inhibited COX-2 expression by 28.3 ± 4.72% and 33.17 ± 4.57%, respectively. Oleoresin did not significantly inhibit COX-2 expression in the present study. Regarding NRF2 expression, none of the tested compounds exhibited a statistically significant difference from the control group (Figure 4).

 Considering the capacity to interact with COX-2, chemoinformatic and *in vivo* investigations suggested the thermodynamically feasible binding of *P*.*emarginatus*-derived vouacapane diterpenoids to both human and murine COX-2,^28^ as well as other pro-inflammatory targets.^35^ Notwithstanding, our results showed that the isolated compounds V2 and V3 inhibited COX-2 expression, while *P*.*emarginatus* oleoresin did not promote the same effect. Although the results are seemingly paradoxical given that the oleoresin contains V2 and V3, literature states that the synergism between compounds in natural products may enhance or hinder their biological activities, which suggests that the compounds in the oleoresin may hinder its COX-2 expression inhibitory effects.^44-46^ In parallel, we did not observe modulation of the NRF2 pathway by *P*.*emarginatus* oleoresin, as well as by vouacapane diterpenoids, demonstrating that these substances do not act through regulation of cell redox status and activation of antioxidant defenses.

###  Cytokine measurement in HaCaT cells exposed to UVA radiation

 Cytokines are noteworthy compounds whose activity is closely related to all processes in an inflammatory reaction. In this context, compounds such as IL-1β, IL- 6, IL-8, IL-10, IL-12p70, and TNF-α may promote diverse responses, ranging from cell migration to apoptosis.^47-49^ Therefore, in order to shed light on the anti-inflammatory properties of *P*.*emarginatus* oleoresin and its main phytoconstituents, pro-inflammatory cytokines were tentatively determined by flow cytometry.

**Figure 5:**
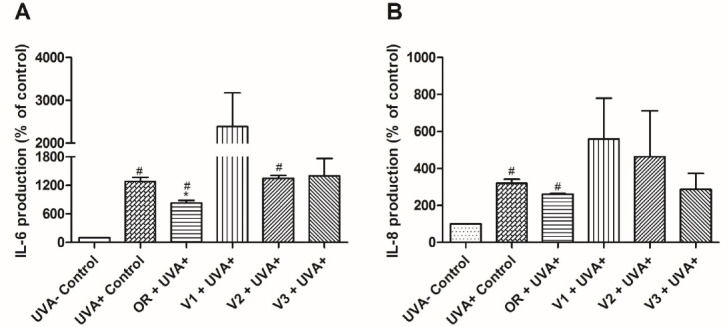
Effects of *Pterodon emarginatus* oleoresin (OR) or isolated vouacapanes on the production of IL-6 (A) and IL-8 (B). Inhibitory effect on IL-6 or IL-8 production in control and tested groups exposed to UVA radiation (20 J/cm^2^) and pre-treated with OR (7.5 µg/mL), V1 (19 µg/mL), V2 (13.8 µg/mL) or V3 (4 µg/mL) for 24 h. Cytokines were quantified using the Cytometric Bead Array method. Each bar represents the mean ± SD of three independent experiments. (^#^*P* < 0.05 vs UVA - control, * P < 0.05 vs. UVA ^+^ control)

 Although the method employed herein is a standard protocol for cytokine determination, TNF-α, IL-10, and IL-12p70 levels were below the detection limit, and IL-1β expression was not altered after exposure to UVA. Therefore, only the IL-6 and IL-8 results are depicted in Figure 5 (A and B).

 The results showed that UVA exposure increased IL-6 and IL-8 expression in HaCaT cells, and the oleoresin inhibited IL-6 expression by 35.47 ± 4.6% in comparison to UVA ^+^ control, while IL-8 did not display statistically significant variation. Furthermore, oleoresin displayed higher inhibitory activity toward IL-6 expression than its isolated diterpenoid compounds (Figure 5A). Although there was no statistical significance, it is interesting to note that V1 and V2 promoted an increase in IL-6 and IL-8 production after exposure to UVA radiation. This probably occurred because of the photoreactivity of V1 and V2, which has not been previously investigated and can be a limitation for evaluation using the proposed model. However, alterations in IL-6 production triggered by oleoresin treatment encouraged further investigation regarding cytokine modulation by *P. emarginatus*.

 IL-6 is an important signaling molecule involved in inflammation and programmed cell death, and its inhibition is thought to promote anti-inflammatory effects. This suggests that the inhibition of IL-6 promoted by oleoresin may play a role in the anti-inflammatory properties of this *P*.*emarginatus*derivative, even though IL-8 did not show statistically significant variation in its inhibition between the tested samples. In this sense, it can be implied that *P*.*emarginatus* oleoresin anti-inflammatory activity involves immunomodulation through IL-6 inhibition.

###  In vitro inhibition of phospholipase A2

**Figure 6:**
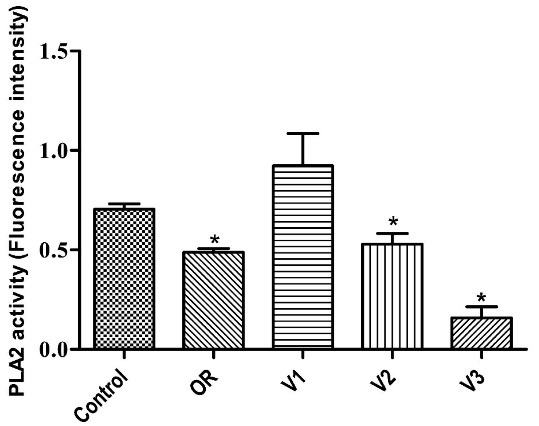
Effects of *Pterodon emarginatus* oleoresin (OR) or isolated vouacapanes on the inhibition of Phospholipase A2 (PLA2). Isolated PLA2 were incubated with OR (7.5 µg/mL), V1 (19 µg/mL), V2 (13.8 µg/mL) or V3 (4 µg/mL) for 10 minutes. PLA2 activity was measured in a fluorimeter, according to instructions of EnzChek Phospholipase A2 Assay-kit. Each bar represents the mean ± SD of three independent experiments. (*P < 0.05 vs. control)

 Given the critical role of phospholipase A2 in inflammation, *P*.*emarginatus* oleoresin and the isolated vouacapane diterpenoids had the capacity to inhibit this macromolecule (Figure 6).

 Oleoresin, V2, and V3 inhibited phospholipase A2 activity by 30.78 ± 2.69%, 24.96 ± 7.72%, and 77.64 ± 8.1%, respectively. However, V1 did not significantly inhibit this enzyme. All comparisons were made using DMSO 0.15% as a control (Figure 6).

 The literature reports that many Fabaceae species have anti-inflammatory diterpenoids whose therapeutic target may include phospholipase A2.^50,51^ This enzyme is responsible for fatty acid cleavage, rendering arachidonic and lysophosphatidic acid, which are nonetheless involved in the metabolic signaling of inflammation.^52^ As the anti-inflammatory properties of the oleoresin are well recognized in Brazilian folk medicine,^53,54^ the significant inhibition of phospholipase A2 by this compound is a remarkable finding. Moreover, given that V2 and V3 also promoted inhibition (V3 being the highest among the isolated compounds), our results suggest that vouacapane diterpenoids are involved in the anti-inflammatory action of *P*.*emarginatus* oleoresin.

###  Pterodon emarginatus oleoresin and isolated vouacapanes as an anti-inflammatory therapeutic approach for COVID-19.

 Since COVID-19 pathophysiology is associated with an inflammatory process that can result in severe damage, the use of anti-inflammatory drugs should be a primary approach.^55^ However, the use of non-steroidal anti-inflammatory drugs (NSAIDs) is still controversial.^56-58^

 Acute respiratory tract infections are associated with an increased chance of stroke and myocardial infarction,^59^ and the use of NSAIDs such as ibuprofen, naproxen, and diclofenac has been associated with higher rates of cardiovascular emergencies, concomitantly with the nature of the infection process.^60,61^ In this context, few options remain to handle the inflammatory effects. Furthermore, NSAIDs cause nephrotoxicity, which is more likely among the patient groups to be severely affected by COVID-19.^56^ Hence, further studies on the effect of anti-inflammatory drugs in clinical use and new therapeutic approaches with anti-inflammatory properties against COVID-19 are pivotal.

 Generally, the principal mechanisms of NSAID action are inhibition of COX-1 and COX-2.^60^ However, the anti-inflammatory effects are due to COX-2 inhibition. COX-2 plays an important role in the inflammatory process triggered by influenza virus infection.^62^ Presumably, a similar process is associated with COVID-19 disease, especially when considering the induction of pro-inflammatory cytokine storms, notably similar to other pathogenic viruses in humans.^63^ COX-2 selective inhibition displays divergent responses in the lung, varying according to kinetics; the recruitment of inflammatory cells into the pleura in 2 hours is limited. However, there is an increase in pleural inflammation within 48 hours.^64^ In addition, the expression of interleukins associated with an adaptive immune response was modulated during the use of NSAIDs, and IL-4 levels were inhibited,^65^ jeopardizing the early production of IFN-γ by innate immune cells, which represent an effective strategy for defense against viruses.^66^ The secretion regulation of this inflammatory mediator is complex and needs to be finely regulated. Our data indicate that the innovative molecules V2 and V3 vouacapanes are competent to inhibit COX-2 expression by 28.3% and 33.17%, respectively. Nonetheless, there was no total inhibition, which could be considered in studies to control exacerbated inflammatory processes, which require adjustments, as seen in the SARS-CoV-2 infection process.

 Interestingly, a meta-analysis showed that IL-6 concentrations were 2.9-fold higher in patients with complicated COVID-19 in comparison with patients with the non-complicated disease.^67^ Therefore, this cytokine may be a prognostic marker to be considered.^68^ Additionally, the use of tocilizumab and its inhibitory effect on IL-6 appears to be an effective and safe approach for a preliminary investigation.^67^ In our study, we observed that oleoresin was able to inhibit IL-6 production by 35.47%, and considering that the cytotoxicity evaluation demonstrated the use of these compounds to be safe, a more in-depth investigation of the possible reducing action of these compounds on the inflammatory effects resulting from COVID-19 and increased IL-6 should be considered.

 An *in vivo* study of other coronaviruses, including SARS-CoV and MERS, demonstrated that age-dependent increase of phospholipase A2 (PLA2) group IID (PLA2G2D) in the lungs contributed to more harmful outcomes in mice infected with severe acute respiratory syndrome-coronavirus.^69^ The authors reported that oxidative stress, via lipid peroxidation, was found to induce PLA2G2D expression in mice and human monocyte-derived macrophages. Therefore, it is rational to presume that guided inhibition of a specific type of phospholipase, such as PLA2G2D, in the lungs of elderly patients with severe respiratory infections, would undoubtedly be a promising therapeutic approach. Considering oleoresin and the vouacapanes V2 and V3 inhibited phospholipase A2 activity by 30.78%, 24.96%, and 77.64%, respectively, and could have a negative role in SARS-CoV-2 clinical outcome (Figure 6), a knowledgeable comprehension of the inhibition mechanism of this phospholipase may, in the future, allow modeling to selectively inhibit a specific class, such as PLA2G2D, which could result in a safe and efficient approach; however, this is still a speculative idea that requires further studies.

 Corroborating our hypothesis of a therapeutic approach, several studies have demonstrated the use of pharmaceutically active natural products as a promising strategy to prevent the worsening of COVID-19. Natural products are well recognized for their antiviral, anti-inflammatory, and immunomodulatory properties. Regarding the inflammatory response, it was observed that the administration of kaempferol reduced serum levels of TNF-α and IL-1β, and that these compounds can block a cation-selective channel expressed in the infected cell by SARS-CoV.^18^ A review showed the applicability of different herbal medicines traditionally used in China, with potential for the treatment of COVID-19-related acute respiratory syndrome. *In vitro* and *in silico* analyses are being carried out, and promising results have been described.^70^

 Moreover, diterpenes have been evaluated not only as anti-inflammatory agents, but also as antiviral agents.^71,72^ The antiviral activity seems to combine two mechanisms: i) protease inhibition, with the interaction ligand-receptor changing the protein conformation and thus stopping virus replication; ii) interference in virus entry into the cell.^72,73^

 Therefore, *P. emarginatus*Vogel has tremendous therapeutic potential, and studies have proven that these terpenes are the main compounds responsible for the biological activity attributed to the species, such as anti-inflammatory and analgesic activities, among others.^31,74^

## Conclusion

 In the current COVID-19 context and its dangerous inflammation, followed by organ failure, the anti-inflammatory properties of *P. emarginatus*oleoresin and isolated vouacapanes reported in this study could offer an attractive therapeutic approach for COVID-19 and post-COVID-19 symptoms. The reduction or chronological control of the inflammatory mediators IL-6, COX-2, phospholipase A2, and INF-γ (indirectly) during the SARS-CoV-2 infection process is an exciting route to be investigated. In addition, since the options of clinical NSAIDs may be at risk of aggravating the patient’s condition, covering treatment methodologies is necessary and desirable.

## Acknowledgments

 CAPES (Coordenação de Aperfeiçoamento de Pessoal de Nível Superior, Brazil - Finance Code 001), FAPDF (Fundação de Apoio à Pesquisa do Distrito Federal), Brasília/DF, and CNPq (Conselho Nacional de Desenvolvimento Cientíﬁco e Tecnológico).

## Author Contributions

**Conceptualization: **Leandra de Almeida Ribeiro Oliveira, Marize Campos Valadares, Maria Tereza Freitas Bara, Dâmaris Silveira.

**Data curation:** Leandra de Almeida Ribeiro Oliveira, Marize Campos Valadares, Dâmaris Silveira.

**Formal Analysis:** Leandra de Almeida Ribeiro Oliveira, Arthur Cristian Garcia da Silva, Fabiana Brandão, Marize Campos Valadares, Dâmaris Silveira.

**Funding acquisition:** Marize Campos Valadares, Maria Tereza Freitas Bara, Dâmaris Silveira.

**Investigation:** Leandra de Almeida Ribeiro Oliveira, Arthur Cristian Garcia da Silva, Douglas Vieira Thomaz, Fabiana Brandão, Marize Campos Valadares, Maria Tereza Freitas Bara, Edemilson Cardoso da Conceição, Dâmaris Silveira.

**Methodology: **Marize Campos Valadares, Arthur Cristian Garcia da Silva, Edemilson Cardoso da Conceição, Maria Tereza Freitas Bara.

**Project administration:** Marize Campos Valadares, Maria Tereza Freitas Bara, Dâmaris Silveira.

**Resources:** Marize Campos Valadares, Maria Tereza Freitas Bara, Dâmaris Silveira.

**Sofware: **ChemDraw 8.0 (Chemical structures); GraphPad Prism 5.0 (Statistics).

**Supervision:** Marize Campos Valadares, Maria Tereza Freitas Bara, Dâmaris Silveira.

**Validation: **Leandra de Almeida Ribeiro Oliveira, Arthur Cristian Garcia da Silva, Fabiana Brandão, Edemilson Cardoso da Conceição, Dâmaris Silveira.

**Visualization:** Leandra de Almeida Ribeiro Oliveira, Arthur Cristian Garcia da Silva, Douglas Vieira Thomaz, Fabiana Brandão, Marize Campos Valadares, Maria Tereza Freitas Bara, Edemilson Cardoso da Conceição, Dâmaris Silveira.

**Writing original draft:** Leandra de Almeida Ribeiro Oliveira, Arthur Cristian Garcia da Silva, Douglas Vieira Thomaz, Marize Campos Valadares, Maria Tereza Freitas Bara, Dâmaris Silveira.

**Writing review & editing:** Leandra de Almeida Ribeiro Oliveira, Arthur Cristian Garcia da Silva, Fabiana Brandão, Marize Campos Valadares, Maria Tereza Freitas Bara, Dâmaris Silveira.

## Ethical Issues

 This research is registered at the Brazilian Sistema Nacional de Gestão do Patrimônio Genético (SisGen), under the number A98042A.

## Conflict of Interest

 The authors declare there is no conflict of interest related to the present work.
